# Supplementary material for: Sequential Cascade Doping of Conjugated-Polymer-Wrapped Carbon Nanotubes for Highly Electrically Conductive Platforms
Source: Polymers (Basel). 2024 Jul 1;16(13):1884. doi: 10.3390/polym16131884 (PMC11244060; doi:10.3390/polym16131884)
Supplement: Supplementary file 1 [file polymers-16-01884-s001.zip › polymers-3058961-supplementary.pdf]

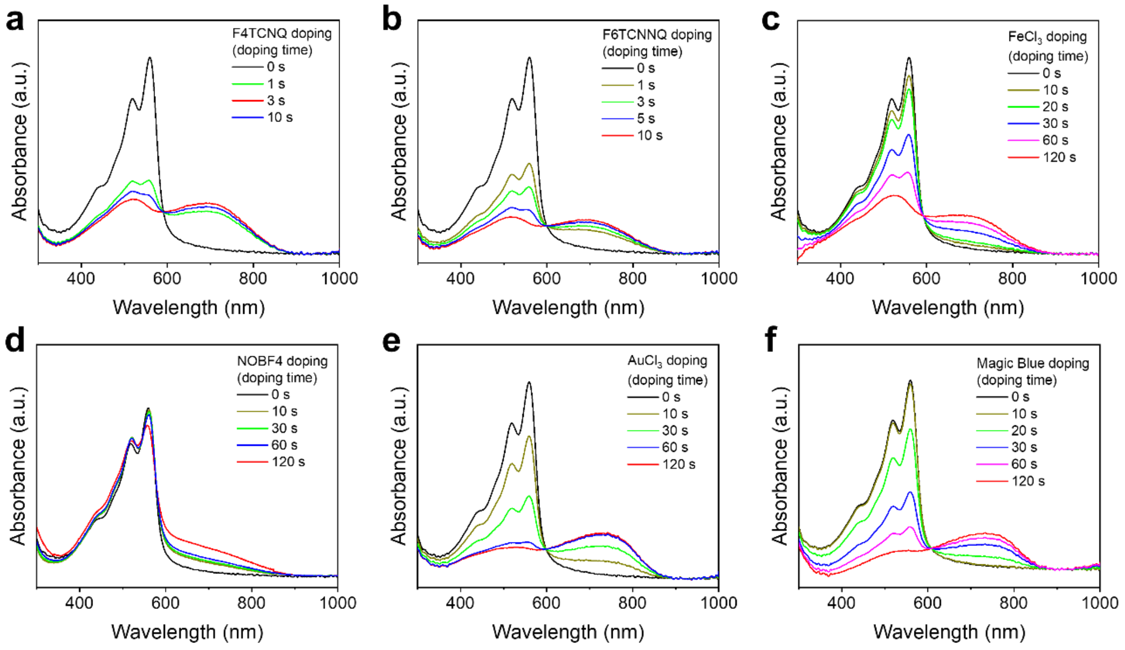

Figure S1. The change in UV-vis absorption spectra of doped PIDF-BT according to doping time for (a) F4TCNQ, (b) F6TCNNQ, (c) $FeCl_3$, (d) NOBF4, (e) $AuCl_3$, and (f) Magic Blue doping.

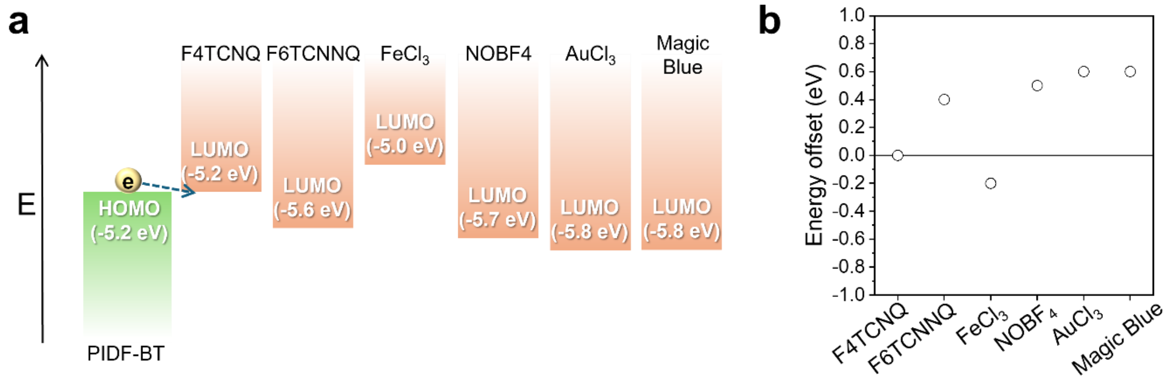

Figure S2. (a) Summary of the energy levels of PIDF-BT and each dopant, and (b) energy level offset between the HOMO of PIDF-BT and the LUMO of each dopant.

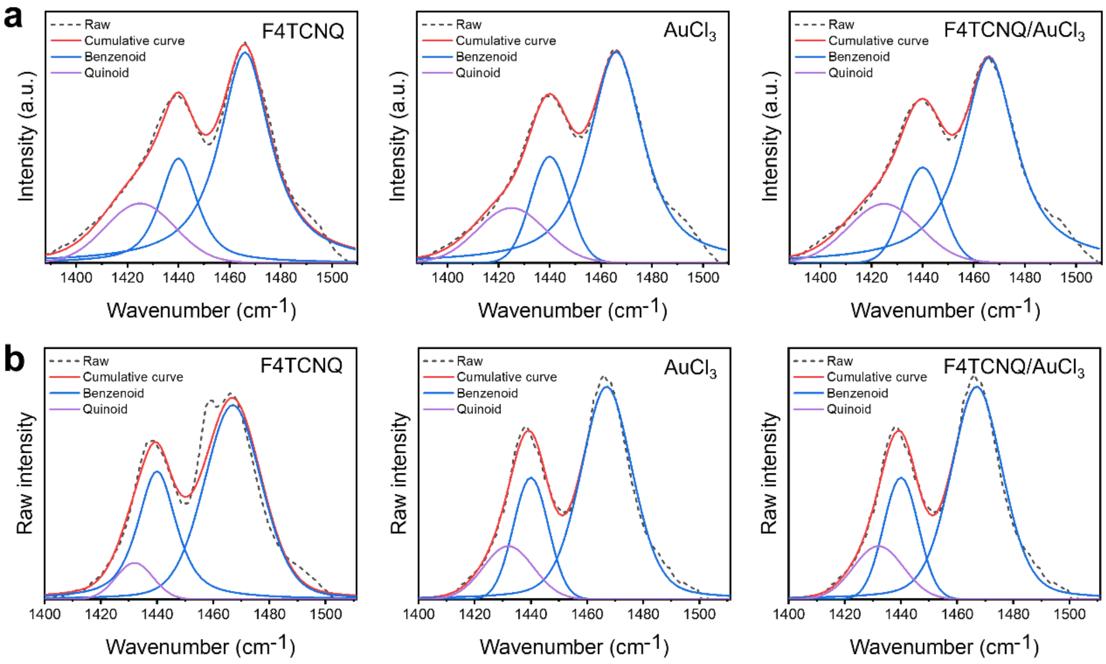

Figure S3. Gaussian fitting of Raman spectra for (a) doped PIDF-BT and (b) doped PIDF-BT@SWCNT
